# Imaging technologies for monitoring the safety, efficacy and mechanisms of action of cell-based regenerative medicine therapies in models of kidney disease

**DOI:** 10.1016/j.ejphar.2016.06.056

**Published:** 2016-11-05

**Authors:** Jack Sharkey, Lauren Scarfe, Ilaria Santeramo, Marta Garcia-Finana, Brian K. Park, Harish Poptani, Bettina Wilm, Arthur Taylor, Patricia Murray

**Affiliations:** a Department of Cellular and Molecular Physiology, Institute of Translational Medicine, University of Liverpool, Liverpool L69 3GE, UK; b Centre for Preclinical Imaging, University of Liverpool, Liverpool L69 3GE, UK; c Department of Biostatistics, Institute of Translational Medicine, University of Liverpool, Liverpool L69 3GE, UK; d Department of Molecular and Clinical Pharmacology, Institute of Translational Medicine, University of Liverpool, Liverpool L69 3GE, UK

**Keywords:** Stem cells, Preclinical imaging, Multispectral optoacoustic tomography, Cell tracking, Biodistribution, Kidney function

## Abstract

The incidence of end stage kidney disease is rising annually and it is now a global public health problem. Current treatment options are dialysis or renal transplantation, which apart from their significant drawbacks in terms of increased morbidity and mortality, are placing an increasing economic burden on society. Cell-based Regenerative Medicine Therapies (RMTs) have shown great promise in rodent models of kidney disease, but clinical translation is hampered due to the lack of adequate safety and efficacy data. Furthermore, the mechanisms whereby the cell-based RMTs ameliorate injury are ill-defined. For instance, it is not always clear if the cells directly replace damaged renal tissue, or whether paracrine effects are more important. Knowledge of the mechanisms responsible for the beneficial effects of cell therapies is crucial because it could lead to the development of safer and more effective RMTs in the future. To address these questions, novel in vivo imaging strategies are needed to monitor the biodistribution of cell-based RMTs and evaluate their beneficial effects on host tissues and organs, as well as any potential adverse effects. In this review we will discuss how state-of-the-art imaging modalities, including bioluminescence, magnetic resonance, nuclear imaging, ultrasound and an emerging imaging technology called multispectral optoacoustic tomography, can be used in combination with various imaging probes to track the fate and biodistribution of cell-based RMTs in rodent models of kidney disease, and evaluate their effect on renal function.

## 1 Introduction

Cell-based regenerative medicine therapies (RMTs) are showing great promise in rodent models of kidney disease (Bussolati and Camussi, 2015, Murray and Woolf, 2014) but clinical translation of these novel therapies is currently hampered because accurate safety and efficacy data from the rodent studies are lacking. These data are essential for determining the risk:benefit ratio of the RMTs in order to judge whether they would be appropriate for use in man. A difficulty in assessing cell-based RMTs is that the standard ‘absorption, distribution, metabolism and excretion’ (ADME) and pharmacokinetic (PK) testing that are used to assess the disposition of pharmacological compounds are not directly applicable. This is mainly because, unlike pharmacological compounds, cellular therapeutics can persist and even proliferate in the recipient over the long-term, and also have the potential to migrate to other tissues where they could cause adverse effects (Heslop et al., 2015). Nevertheless, the general scientific principles in the fields of pharmacology and toxicology should be considered and applied where possible. The application of these principles is facilitated by recent progress in the field of in vivo imaging, which is making it possible to visualise administered stem cells, track their fate and ‘see’ the effects they have on host tissues and organs (James and Gambhir, 2012, Meleshina et al., 2015, Wang and Yan, 2008), thus enabling the behaviour of administered cells to be evaluated with a degree of accuracy that until now, has only been possible for drugs. For instance, using the appropriate imaging agent/imaging modality combination, it is possible to determine how an administered cell population is distributed within each body compartment, thus defining the maximum tissue distribution (equivalent to ‘Cmax’ for administered drugs). Then by measuring the distribution kinetics of the cells, it is possible to define the complete spatiotemporal profile of distribution (equivalent to ‘pharmacokinetics’ (PK) for administered drugs) and the rate of accumulation and elimination from target and non-target tissues. Simultaneously, it is also possible to monitor the biological effects on host tissues and organs, thus defining the complete spatiotemporal profile of responses (equivalent to ‘pharmacodynamics’ (PD) for administered drugs). By co-registering and correlating the kinetics and dynamics, it should be possible to define the efficacy and safety for each cell therapy. In this review, we will discuss how in vivo imaging technologies can be used to evaluate cell-based RMTs in rodent models of kidney disease, with particular focus on the biodistribution of cell-based RMTs and their effect on renal function.

## 2 Rodent models of kidney disease

Most studies investigating the potential of cell-based RMTs to treat kidney disease have used rodent models of ischaemia reperfusion injury (IRI) (Donizetti-Oliveira et al., 2012, Feng et al., 2016) or various drug-induced injury models, such as cisplatin, adriamycin, aristolochic acid (Bruno et al., 2012, Li et al., 2012, Qi and Wu, 2013, Ronconi et al., 2009) and the glycerol model of induced rhabdomyolysis (Angelotti et al., 2012, Geng et al., 2014). All of these models are clinically relevant. For instance, IRI, which has been proposed to be the optimal model for evaluating cell-based RMTs (Wang et al., 2012), represents the type of tubular injury incurred by renal allografts during transplantation (Asderakis et al., 2001), and by the kidneys of patients undergoing cardiopulmonary bypass surgery (Okusa et al., 2009). Clinical trials have already been undertaken to assess the potential of mesenchymal stem/stromal cells (MSCs) to ameliorate kidney disease in cardiac surgery patients, with both positive and negative outcomes being reported (NCT00733876; NCT01602328)(Gooch and Westenfelder, 2016). A clinical trial is also currently underway to establish the safety and feasibility of administering MSCs to cancer patients receiving cisplatin (NCT01275612), an anti-cancer drug that causes acute tubular injury, which in 20% of patients, progresses to chronic kidney disease (Inai et al., 2013). Likewise, the safety and efficacy of bone marrow-derived mononuclear cells are being assessed in patients with focal segmental glomerulosclerosis (NCT02693366), a disease that resembles adriamycin-induced nephropathy in rodents (Scarfe et al., 2015). Cell-based therapies for treating aristolochic acid and rhabdomyolysis-induced nephropathy have only been tested in rodent models so far, but both models are good representations of the tubulo-interstitial injury that can occur in human patients following ingestion of aristolochic acid (Yang et al., 2014) or crush injury (Gibney et al., 2014), respectively.

A common problem with all rodent kidney injury models is that the extent of injury incurred can vary considerably between individuals within the same cohort, making it difficult to accurately assess the efficacy of the cell therapies. Some studies address this by using large numbers of animals in the treatment and control groups, and culling animals at various time points (Angelotti et al., 2012, Ronconi et al., 2009). However, an alternative approach is to use methodologies that enable the same animal to be evaluated over time, so that the extent of injury and therapeutic response can be monitored in each individual animal. The key advantage of undertaking such longitudinal assessments is that correlated data are generated, thus increasing the power of the statistical tests, which in compliance with the principles of ‘Replacement, Refinement and Reduction’ (the ‘3Rs’), enables the number of animals in these type of experiments to be reduced.

## 3 Cell-based regenerative medicine therapies

The most common cell types used as RMTs include MSCs from bone marrow (Qi and Wu, 2013) and adipose tissue (Donizetti-Oliveira et al., 2012), kidney-derived progenitor cells (Ronconi et al., 2009), renal progenitors derived from embryonic stem cells or induced pluripotent stem cells (iPSCs) (Toyohara et al., 2015), or heterogeneous populations such as adipose-derived regenerative cells (Feng et al., 2010) or bone marrow-derived mononuclear cells (Semedo et al., 2010). MSCs, adipose-derived regenerative cells and bone marrow-derived mononuclear cells ameliorate renal injury via paracrine factors, whereas kidney-derived progenitor cells have been reported to engraft in the kidney and generate specialised renal cells (Angelotti et al., 2012, Bussolati et al., 2005, Ronconi et al., 2009). iPSC-derived renal progenitors can also engraft in the kidney and generate renal cells (Imberti et al., 2015, Toyohara et al., 2015), though their therapeutic effects appear to be mediated by paracrine mechanisms (Toyohara et al., 2015). As an alternative to administering cells, several studies have investigated the therapeutic potential of cell-derived extracellular vesicles, which in many cases, have been shown to be as efficacious as the cells themselves (Bruno et al., 2009). It is anticipated that extracellular vesicles would be less hazardous than cells as they would not form tumours and would present a low risk of forming emboli. As we will discuss in Section 5, it is crucial to monitor the in vivo biodistribution of cellular therapeutics in order to assess their safety, efficacy and mechanisms of action. There are two broad methods for labelling cells so that they can be tracked following their administration:introducing a genetic reporter, or labelling the cells with a nanoprobe or small molecules, such as near infrared (NIR) dyes or fluorescent proteins. For adipose-derived regenerative cells and bone marrow-derived mononuclear cells, which are heterogeneous populations of autologous cells that are used at the point-of-care, it is not possible to introduce genetic reporters, because this would require culturing the cells in vitro, a process which would be expected to alter their composition and phenotype. MSCs, iPSCs and kidney-derived progenitor cells on the other hand, are routinely expanded in vitro, and so for these cell types, there is the opportunity to introduce reporters. The biodistribution of extracellular vesicles can be monitored using both genetic reporters and NIR dyes (Grange et al., 2014b, Lai et al., 2014).

## 4 Imaging agents and technologies

### 4.1 Imaging agents for cell tracking

Genetic reporters are excellent tools for tracking cell fate and biodistribution in small animals. When expressed under the control of a constitutive promoter, reporter genes can be used for long-term biodistribution analysis, as the signal is not depleted when the cells proliferate. Constitutively expressed reporters also indicate whether the cells are viable, because expression is rapidly lost if the cells die. When expressed under the control of a cell-type specific promoter, reporters can be used to monitor cell fate and/or function by indicating the differentiation status of administered cells. The most commonly used reporter for cell tracking studies is firefly luciferase, an enzyme that emits light in the presence of D-luciferin, oxygen and ATP and can be detected using bioluminescence imaging. Other luciferases include the sea pansy (*Renilla reniformis*) and marine cope pod luciferases (*Gaussia princeps*), but compared to firefly luciferase, the *Renilla* is less intense, and the *Gaussia* has a very short emission half-life (James and Gambhir, 2012). In addition to bioluminescence imaging, genetic reporters can also be used for imaging with other modalities; for instance, NIR fluorescent protein reporters can be used for fluorescence (Lu et al., 2013) and photoacoustic imaging (Jathoul et al., 2015), and cells expressing nuclear imaging reporters, such as the human norepinephrine transporter, can be imaged with single photon emission computed tomography (SPECT) following administration of an appropriate substrate (e.g., ^123^I-MIBG; meta-iodo-benzylguanine) (Moroz et al., 2007). There has also been some interest in using reporter genes for magnetic resonance imaging (MRI) (Velde et al., 2013), but the low sensitivity of MRI reporters means they have limited use in cell tracking applications (Pereira et al., 2016a, Pereira et al., 2015, Pereira et al., 2016b).

In addition to genetic reporters, nanoparticles and small molecules such as NIR dyes are also useful tools for tracking the biodistribution of administered cells (Taylor et al., 2012). Unlike the reporter genes, they cannot be used to monitor cell fate, and due to them being depleted by 50% with each cell division, they are not suitable for tracking proliferating cells in the long-term. Furthermore, if the labelled cell dies, they can be taken up by host cells, leading to false positive results (Taylor et al., 2012). However, a key advantage of these non-genetically encoded imaging probes is that in most cases, very high labelling efficiencies can be achieved (typically over 95%) following relatively short incubation times (4–24 h) (Taylor et al., 2014). Moreover, with the exception of the luciferases, much higher signal intensities can be obtained than with genetic reporters, making it possible to detect fewer numbers of cells. There are a wide range of different types of non-genetically encoded imaging probes, enabling cells to be tracked with all the major imaging modalities. For instance, superparamagnetic iron oxide nanoparticles (Taylor et al., 2012) and fluorine (19F)-based imaging agents (Tirotta et al., 2014) are used for MRI; gold nanorods and NIR dyes for photoacoustic imaging; NIR dyes for fluorescence imaging; persistent luminescent particles for bioluminescence imaging (Maldiney et al., 2014); technetium (^99m^Tc) for SPECT; ^18^F-fluorodeoxyglucose (^18^F-FDG) for positron emission tomography (PET) (Rosado-de-Castro et al., 2014); and perfluorocarbon nanoparticles for ultrasound imaging (Winter, 2014).

### 4.2 Imaging technologies for tracking cells and monitoring their effects on host tissues

**Fig. 1:**
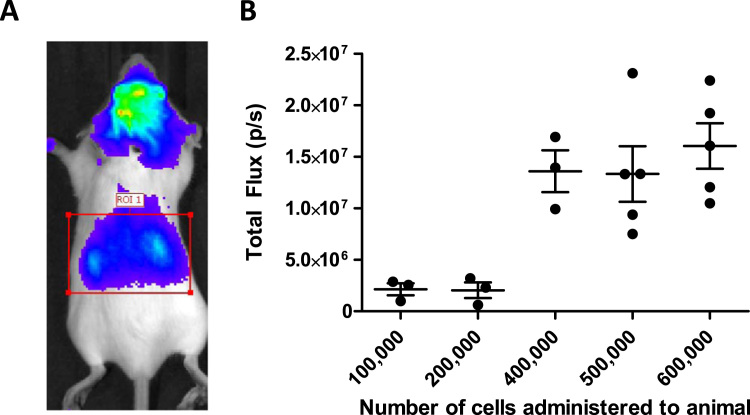
Radiance in the abdominal region of mice that received an intra-cardiac injection of luciferase+ cells does not increase linearly. Mouse kidney-derived stem cells were administered into the left cardiac ventricle in the range 1×105 to 6×105 and imaged immediately using BLI (IVIS Spectrum; Perkin Elmer). A region of interest (ROI) was drawn in the same position on each animal as shown in (A), and the total Flux recorded in (B).

**Fig. 2:**
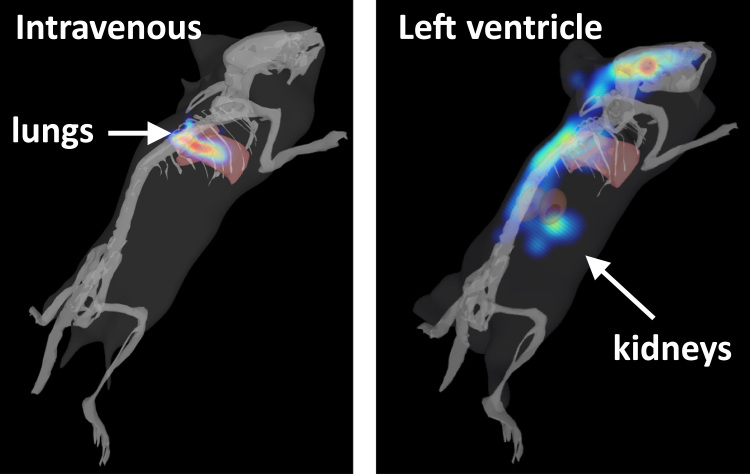
Whole-body biodistribution of luciferase+ cells using 3D diffuse light imaging tomography. Human kidney-derived cells expressing luciferase were administered either intravenously or into the left cardiac ventricle of healthy mice and imaged immediately using a bioluminescence imager (IVIS Spectrum, Perkin Elmer). Following IV administration, cells are located in the lungs, and following intra-cardiac administration, some cells are located in the kidneys.

The following imaging technologies can be used for cell tracking and assessing the effects of the cells on host tissues in small animals: MRI, nuclear imaging (i.e., SPECT and PET), ultrasound, fluorescence, bioluminescence and photoacoustic imaging. However, all of these modalities have some limitations (James and Gambhir, 2012). For instance, MRI offers excellent spatial resolution, but temporal resolution is poor, so while organ-focussed imaging is possible, performing whole body scans is not really feasible. Nuclear imaging techniques permit whole body scanning and generate quantitative data, but suffer from poor spatial resolution, and perhaps more importantly, require animals to be exposed to ionising radiation, which is particularly problematic for longitudinal studies that necessitate repeated scanning. Ultrasound imaging is safe, but mainly gives structural, rather than molecular information, though when used in combination with microbubble contrast agents, it can be very useful for monitoring renal perfusion (Mahoney et al., 2014). Fluorescence imaging is also safe, but sensitivity is poor and there is significant signal attenuation with increasing depth, so that ~100,000 cells emitting NIR fluorescence would be required in an internal organ such as the kidney in order to generate a detectable signal. Bioluminescence imaging is safe and has much greater sensitivity than MRI and fluorescence, allowing fewer than 100 cells to be detected, but because the strength of the emitted signal is affected by various parameters, including tissue depth and substrate availability (luciferin in the case of firefly luciferase), it can be difficult to acquire reliable quantitative data in some applications (Fig. 1). An emerging imaging technology known as photoacoustic imaging overcomes many of the limitations of the aforementioned modalities. For instance, it has excellent sensitivity, allowing small numbers of cells to be detected; spatial and temporal resolution are both very good, permitting rapid whole body scanning of small rodents; it can generate quantitative data; it is completely safe, allowing repeated scanning; and for small animals such as mice, a particular type of photoacoustic scanner known as ‘multispectral optoacoustic tomography’ (‘MSOT’, built by iThera Medical Ltd) permits the entire depth of a mouse to be imaged without signal attenuation (Taruttis and Ntziachristos, 2015). However, a draw-back with MSOT (and all other photoacoustic scanners) is that visualisation of the lungs is not possible due to the presence of air in this organ. This is an important issue for cell tracking because it is known that most cell types tend to become trapped in the lungs following intravenous administration (Fischer et al., 2009, Tögel et al., 2008) and Fig. 2.

Although no single imaging technology/imaging agent is capable of providing the breadth of information required, by using multimodal strategies that combine different imaging technologies, it is possible to monitor the biodistribution and fate of administered cells while simultaneously evaluating the effects on the tissues the cells populate. For instance, the easiest and most useful strategy for monitoring the whole-body biodistribution of cells in small animals is to introduce the firefly luciferase reporter and undertake bioluminescence imaging. However, because the spatial resolution of bioluminescence imaging is low and only a planar image is generated, it is difficult to pinpoint the exact location of the cells on the z-axis. Although locating the cells on the z-plane can be partially addressed by using 3D diffuse light imaging tomography (Fig. 2), bioluminescence imaging does not allow the intra-renal biodistribution of the cells to be monitored. However, this could be addressed by labelling the luciferase^+^ cells with either superparamagnetic iron oxide nanoparticles or gold nanorods, and then undertaking bioluminescence imaging followed immediately by MRI or MSOT, respectively.

## 5 Biodistribution of RMTs in kidney disease models

### 5.1 Safety, efficacy and mechanisms of action

To assess the safety of cell-based RMTs, knowledge of the biodistribution of the cells is required so that any potential adverse effects on the tissues the cells populate can be monitored, the most common potential adverse effects being embolism, inflammation, fibrosis, immunogenicity, mal-differentiation and tumourigenesis (Heslop et al., 2015). The risk of any particular adverse effect is dependent on the cell type. Larger cell types are more likely to be entrapped in capillary beds and thus pose an increased risk of emboli formation (Fischer et al., 2009), whereas pluripotent cells have a greater propensity to form tumours. Particular care is needed with MSCs, which in some environments, can readily differentiate to form osteoblasts, chondrocytes and/or adipocytes, as shown in a previous study where MSCs administered in a rat IRI model generated adipocytes within the glomeruli, impairing renal function in the longer term (Kunter et al., 2007). Biodistribution studies are also necessary to assess the efficacy of cell therapies; for instance, it is important to know what proportion of the administered cell population reaches the kidneys, and for how long they persist, so that the relationship between efficacy and the intra-renal distribution of the cells can be determined. This information can give valuable insight into the mechanisms of action of the cells, as illustrated by a recent study by Geng et al. (Geng et al., 2014) which showed that MSCs can ameliorate renal injury in a mouse rhabdomyolysis model despite them not engrafting in the kidney. Follow-on experiments suggested that the MSCs reduced kidney injury in this case by inducing endogenous macrophages to adopt an M2-like (i.e., ‘anti-inflammatory) phenotype.

### 5.2 Renal engraftment and the route of administration

Intravenous (i.v.) administration is the most commonly used route for administering cells in rodent models of kidney injury. Although it is well-documented that most cell types, including MSCs, become entrapped in the pulmonary capillaries following i.v. administration (Fischer et al., 2009), some reports suggest that MSCs (Morigi et al., 2010) and kidney-derived progenitor cells (Ronconi et al., 2009) can bypass the pulmonary circulation and engraft in the kidney. It is important to note, however, that studies reporting renal engraft of cells following i.v. administration typically use the lipophilic dye, PKH26, to identify cells in histological sections of renal tissue, rather than using in vivo imaging approaches. The problem with lipophilic dyes is that they are readily transferred to host cells, leading to false positive results (Agrawal et al., 2014, Lassailly et al., 2010, Progatzky et al., 2013). This problem is compounded by the fact that renal tissue emits high levels of autofluorescence, with levels being increased even further following injury (Sun et al., 2011). Hence, there is a risk that some of the patchy fluorescence that appears in damaged renal tissue could be mistaken for cells labelled with PKH26 or other fluorescent markers. Nevertheless, there are some reports that do appear to show the presence of small numbers of cells in the kidney following i.v. administration (Grange et al., 2014a). An explanation for this could be that certain nephrotoxic agents (e.g., glycerol-induced rhabdomyolysis) also cause acute lung injury, with the resulting hypoxia (Rodrigo et al., 2006) potentially leading to the recruitment of intrapulmonary arteriovenous anastomoses (IPAVAs) (Bates et al., 2012). IPAVAs are large diameter vessels in the lung that directly connect the arterial and venous networks, thus bypassing the pulmonary capillaries (Lovering et al., 2010). Interestingly, if 10^6^ 15 µm diameter fluorescent microspheres (approximately the same diameter as mouse MSCs) are injected into the superior vena cava of rats under normoxic conditions, no beads are observed in the kidneys, whereas under hypoxic conditions, approximately 10^3^ beads are detected (Bates et al., 2012). Each kidney receives ~10% of the cardiac output, so if 10^6^ beads were injected into the left cardiac ventricle, it would be expected that 10^5^ would reach the kidneys. The fact that 10^3^ beads are detected following i.v. administration in hypoxic rats shows that under these conditions, ~1% of injected beads can reach the kidneys. By extrapolation, this study suggests that if cells are administered i.v. into hypoxic rodents, it is possible that 1% could engraft in the kidneys. This could explain why in some studies, cells can be observed in the kidneys following i.v. administration, but not in others. However, the small numbers of engrafting cells strongly suggests that the observed therapeutic effects are likely to be mediated by paracrine factors released from the remaining ~90% of the cell population that is entrapped within the lungs.

To circumvent the ‘problem’ of lung entrapment, cells can be delivered into the kidney by administering then on the arterial side of the circulation. In rats, cells can be administered via the renal artery, whereas in mice, the cells need to be administered via the suprarenal aorta (Tögel et al., 2008) or left cardiac ventricle (Fig. 2), due to the renal artery being too small. Following administration of luciferase^+^ MSCs into the suprarenal aorta, bioluminescence imaging showed that MSCs were initially present in the kidneys, but by 24 h, were mainly located in the lung (Tögel et al., 2008). Similar results were obtained following administration of human MSCs into the left cardiac ventricle, where histological analysis showed that cells were observed in the kidneys shortly after being injected, but by 4 weeks, were barely detectable (Bentzon et al., 2005). Likewise, following administration of adipose-derived regenerative cells into the renal artery of rats, cells were initially present within the glomerular capillaries but had almost disappeared by 72 h (Feng et al., 2010). Of note, a study by Zhuo et al. (Zhuo et al., 2013) showed that it luciferase^+^ MSCs were injected into the right renal artery of rats subjected to IRI, bioluminescence was mainly observed in the lung. This is an interesting observation because it suggests that even when cells are injected into the renal artery, the majority exit via the renal vein and become entrapped in the lung. Analysis of renal function and histology in this rat IRI model showed that the therapeutic effects of the MSCs were similar, irrespective of whether they were administered i.v. or via the renal artery, which in light of the biodistribution data, is perhaps not too surprising (Zhuo et al., 2013). Consistent with these findings, a recent meta-analysis shows that cell-based RMTs administered either i.v. or via the renal artery are similarly efficacious in rodent models of chronic kidney disease (Papazova et al., 2015). Moreover, the extent of the therapeutic response appears to be independent of cell dose and the number of administrations.

Cell therapies can also be introduced into the kidney by direct injection into the parenchyma (Harari-Steinberg et al., 2013, Toyohara et al., 2015) or by injecting under the renal capsule (Toyohara et al., 2015), though the invasive nature of these administration routes means they would have little clinical utility. Toyohara et al. showed that although iPSC-derived renal progenitors could integrate into renal tubules following parenchymal injection, the cells did not ameliorate injury in a mouse IRI model. Conversely, following administration under the renal capsule, the cells did not integrate into tubules but could promote renal recovery (Toyohara et al., 2015). These interesting results could possibly be explained by the fact that the subcapsular region of the kidney is a permissive environment that can support the growth of various cell types, including pancreatic islets and pluripotent cells (teratoma assays). Thus, the improved therapeutic efficacy observed following subcapsular administration might have simply been due to the fact that the iPSC-derived renal progenitors could survive for longer in this environment. In support of this, a more recent study has shown that if MSCs are injected into the renal parenchyma within a chitosan-based hydrogel that supports their survival, improved therapeutic efficacy is observed in a mouse model of IRI (Feng et al., 2016).

### 5.3 Whole-body biodistribution of cell-based RMTs in rodent models of kidney disease

A number of studies have shown that renal engraftment of cell-based RMTs increases in some models of kidney injury (Tögel et al., 2008, Grange et al., 2014b). For instance, intra-arterial administration of MSCs in a mouse IRI model leads to a short-term increase in the numbers of cells in the kidneys of injured mice compared with sham-operated controls (Tögel et al., 2008). This increased ‘homing’ could either be due to the injured renal tissue secreting chemo-attractants, or could simply result from the MSCs getting temporarily stuck in the glomerular capillaries because of the decrease in capillary perfusion that typically occurs following ischaemic injury (Jerome et al., 1994).

**Fig. 3:**
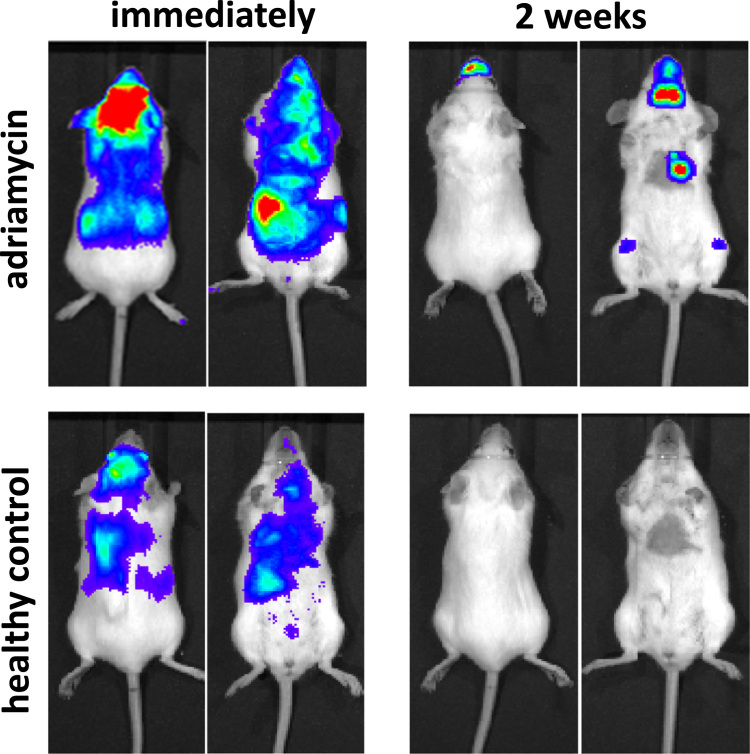
Whole-body biodistribution of luciferase^+^ mouse kidney-derived stem cells using bioluminescence imaging. Cells were administered into the left cardiac ventricle on the 2nd day following IV injection of adriamycin or saline (healthy control) and mice were imaged immediately or 2 weeks later using a bioluminescence imager (IVIS Spectrum, Perkin Elmer). Mice that received adriamycin showed engraftment of cells in regions corresponding to the heart and femoral bone marrow, but not in the kidneys. No cells were detected in control mice at this time point.

Monitoring the whole-body biodistribution of cell therapies is particularly important in models where the nephrotoxic agent damages non-renal tissue as well as the kidneys. This is because cell-based RMTs can readily engraft in various types of injured tissue, as shown in a study where MSCs injected into the aortic arch proliferated in a region of the mouse hind limb that had been damaged with radiation (Kean et al., 2013). Examples of such models include glycerol-induced rhabdomyolysis, which causes damage to the injected muscle and lungs, and adriamycin, which damages the heart and bone marrow (To et al., 2003). Indeed, intra-vital microscopy showed that MSCs labelled with a red fluorescent protein could be detected in the lung and muscle in a mouse rhabdomyolysis model (Geng et al., 2014). Likewise, work from our own laboratory shows that following administration of luciferase^+^ mouse kidney-derived stem cells (Fuente Mora et al., 2012) in a mouse model of adriamycin-induced nephropathy, cells engrafted in areas corresponding to the location of the heart and femoral bone marrow, but not in the kidneys (Fig. 3).

## 6 Monitoring the effect of RMTs on renal function

The glomerular filtration rate (GFR) is the most accurate measure of renal excretory function, but obtaining the GFR requires repeated blood and/or continuous urine sampling over a prolonged period (5–24 h), which is technically challenging in rodents, particularly in mice, where blood is usually only taken following animal sacrifice via cardiac puncture. For this reason, an ‘estimated’ GFR based on levels of serum creatinine is typically used. However, a problem with using serum creatinine measurements is that between 35% and 50% of creatinine is excreted via tubular secretion in rodents rather than glomerular filtration (Eisner et al., 2010), making it a poor predictor of GFR. This problem can be addressed by using a novel electronic device that can give an accurate indication of GFR in rodents by measuring the half-life of intravenously administered FITC-sinistrin (Schock-Kusch et al., 2011), a molecule that is exclusively filtered by the glomeruli. This device has recently been used to monitor changes in GFR in a mouse adriamycin model, where the measurements showed a strong positive correlation with the extent of glomerular histological damage (Scarfe et al., 2015). However, while the transcutaneous device allows longitudinal GFR measurements to be obtained, it does not provide any anatomical information, and cannot be applied to models where only one kidney is injured, as the device measures the global GFR, and does not give a measurement for each individual kidney. Whole animal imaging technologies are therefore essential for undertaking the longitudinal studies required to monitor disease progression and therapeutic responses in the same animals over time. This approach is far superior to sacrificing animals at set time points and undertaking histological analyses, because apart from reducing animal numbers, the longitudinal data obtained offers the opportunity to observe patterns of change at an individual level, as well as increasing the statistical power of the experiment. MRI, nuclear imaging techniques (SPECT and PET) and ultrasound can all be used to monitor the efficacy of cell-based RMTs, but the multiplexing capability of MSOT offers unprecedented opportunities to monitor various aspects of renal function simultaneously, and will thus be discussed in more detail.

### 6.1 MRI, SPECT, PET and ultrasound

**Fig. 4:**
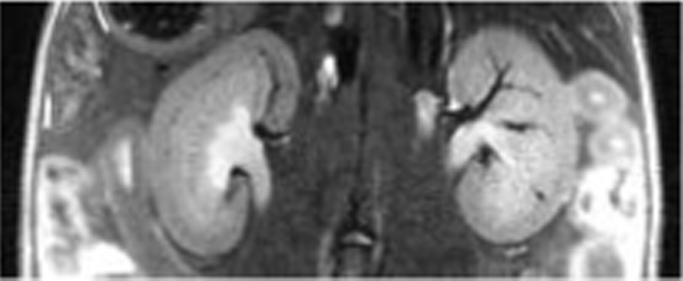
T2-weighted MR scan of the kidneys of a healthy mouse imaged in vivo using a Bruker 9.4 T MR scanner.

Of all the in vivo imaging modalities, MRI gives the highest spatial resolution and is therefore the modality of choice for performing anatomical imaging of the kidney (Fig. 4). As kidney disease progresses, morphological changes occur in the renal parenchyma, which can be monitored using 3D rendering to assess organ volume changes (Zöllner et al., 2013), and with diffusion weighted imaging to monitor changes in renal microarchitecture (Ebrahimi et al., 2013). MRI can also be used to monitor various aspects of renal function, such as renal perfusion and the GFR (using dynamic contrast enhanced MRI), as demonstrated in rat models of adriamycin-induced kidney injury and uninephrectomy, respectively (Egger et al., 2015, Zöllner et al., 2013).

The nuclear imaging techniques, SPECT and PET, do not give any anatomical information and thus require co-registration with MRI or computed tomography (CT), but can be extremely useful for assessing renal function (Durand et al., 2011). For instance, the SPECT tracer, technetium-99 m-mercaptoacetyltriglycine (^99m^Tc-MAG3), is routinely used in the clinic to monitor tubular secretion, and can also be applied to small rodents, as shown in a study where this strategy was used to monitor renal function over time in a mouse model of unilateral IRI (Herrler et al., 2012). SPECT can also be used to monitor GFR by measuring the clearance of the glomerular tracer, ^99m^Tc- diethylenetriamine penta-acetate (DTPA), which has been used to investigate the renoprotective effects of oestrogen in a rat model of ureteric obstruction (Mao et al., 2014). More recently, a novel PET tracer, 2-deoxy-2-^18^F-fluorodeoxysorbitol (^18^F-FDS) has been developed, which like ^99m^Tc-DTPA, can be used to monitor GFR (Wakabayashi et al., 2016). The advantage of PET over SPECT is that in the clinical setting, it offers higher spatial and temporal resolution, enabling more accurate quantitative data to be obtained.

Ultrasound is routinely performed in the clinic to assess renal morphology, and by using Doppler ultrasound, it is possible to monitor renal perfusion (To et al., 2003). The availability of small animal ultrasound scanners now makes it possible to assess renal morphology and function longitudinally in rodents. For instance, by undertaking contrast enhanced ultrasound with microbubble contrast agents, it is possible to monitor regional blood flow longitudinally in the mouse kidney (Sullivan et al., 2009). This technique has been used to monitor renal microperfusion in a mouse IRI model, and to assess the changes in perfusion that occur in the outer medulla over time (Fischer et al., 2016).

### 6.2 MSOT

**Fig. 5:**
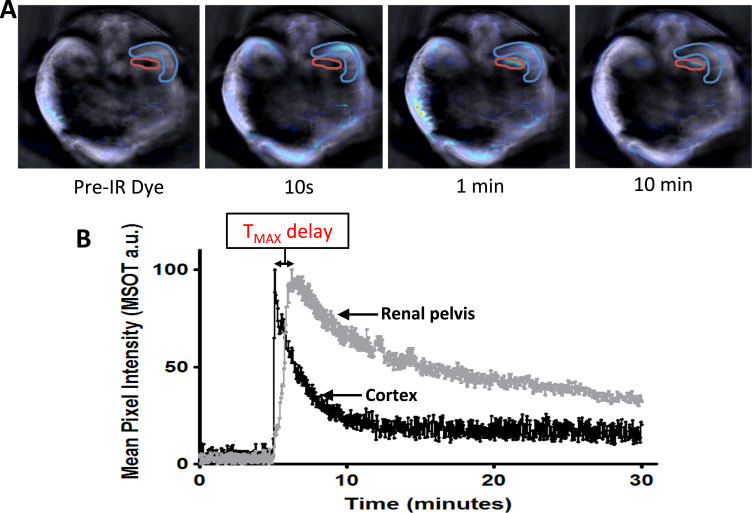
(A) MSOT images showing the cross-section of a healthy mouse prior to and post administration of IRDye800 carboxylate (20 nmol). Blue and red regions of interest represents the renal cortex and pelvis, respectively. The dye is present in the cortex at the 10 s time point, and by 1 min, starts to accumulate in the pelvis. By 10 min, the dye has cleared from the cortex. (B) Graph showing the accumulation and clearance of IRDye800 carboxylate from the cortex and pelvis. (For interpretation of the references to color in this figure legend, the reader is referred to the web version of this article.)

Multispectral optoacoustic tomography (MSOT) is a technique which relies on the photoacoustic effect to facilitate the volumetric and quantitative visualisation of tissues in vivo without the necessity for contrast agents. A laser is used to pulse light of multiple wavelengths towards a target tissue or organ, permitting imaging at high spatial resolution (150 µm) to a depth of ~3 cm. This light is absorbed by endogenous photo-absorbers within the target tissue, which undergo thermoelastic expansion to generate sound waves that are detected by acoustic detectors (Mandal et al., 2015). A particular advantage of MSOT over other imaging modalities is its multiplexing capability, which arises from the ability of the scanner to distinguish different absorbance spectra, enabling several molecular targets to be detected simultaneously. Oxyhaemoglobin and deoxyhaemoglobin are particularly strong intrinsic absorbers that can be readily distinguished by MSOT (Buehler et al., 2010; Wang and Hu, 2012), and could thus provide valuable information on renal perfusion, vascularisation and oxygenation, as has recently been undertaken with tumour tissue (Ermolayev et al., 2016). MSOT not only allows the visualisation of endogenous molecules, but also the simultaneous imaging of exogenous NIR dyes, proteins and nanoparticles such as gold nanorods, which absorb light in the NIR region of the spectrum (Deliolanis et al., 2014). MSOT can acquire cross-sectional images of anaesthetised small animals that can be taken in sequential steps to build a 3D image of the target tissue or organ. Alternatively, one cross-sectional plane can be acquired for the duration of the imaging session to permit the fast dynamic scanning required for real-time pharmacokinetic analyses. This can be utilised to analyse the accumulation and/or clearance of exogenous NIR tracers in different regions of the kidney, enabling different aspects of renal function to be monitored (Fig. 5). In a recently published study by Scarfe et al. (Scarfe et al., 2015), MSOT was used to monitor renal function longitudinally in a mouse adriamycin model. This was achieved by measuring the clearance of IRDye 800 carboxylate, an NIR dye that is rapidly and exclusively excreted by the kidneys (Taruttis et al., 2012). The Scarfe study showed that the time between the mean peak pixel intensity in the cortex and the pelvis (T_MAX_ delay) was significantly greater in mice with adriamycin-induced nephropathy than in healthy mice. Furthermore, the T_MAX_ delay correlated strongly with glomerular scarring, as determined by histological analysis. The multiplexing capability of MSOT means that if the appropriate NIR tracers were available (i.e., tracers that were either exclusively filtered or secreted), it would be possible to assess these two important aspects of renal function simultaneously. In conclusion, MSOT is a tool which has a range of applications for assessing RMTs in kidney disease models: due to its high spatial resolution it can be used to assess renal morphology; it can indicate the oxygenation status of the kidney; it can be used to track the biodistribution and fate of labelled cells and extracellular vesicles; and by monitoring the pharmacokinetics of renally excreted NIR dyes, it can accurately assess renal function.

## 7 Concluding remarks

The development and application of in vivo imaging strategies to accurately assess the safety, efficacy and mechanisms of action of cell-based RMTs will lead to a better understanding of their potential hazards and therapeutic benefits, thus underpinning the safe introduction of these new therapies into the clinic. Indeed, in vivo imaging approaches are already providing novel insights into the mechanisms of action of RMTs in rodent models of kidney disease, which is likely to lead to safer and more effective therapies in the future. For instance, we now know that for the majority of therapeutic cell-types, the regenerative effects on host renal tissue are mediated by paracrine or endocrine factors. Therefore, if these factors could be defined, it could be possible to administer them instead of the cells, thus bypassing some of the potential hazards associated with cell administration. Although the focus of this review has been on renal cell-based RMTs, it is worth noting that in rodent models of various other diseases, including heart disease (Malliaras and Marban, 2011) and spinal cord injury (De Paul et al., 2015), there is increasing evidence that the therapeutic effects are mediated by paracrine factors rather than by the cells themselves.

## References

[bib1] Agrawal H., Shang H., Sattah A.P., Yang N., Peirce S.M., Katz A.J. (2014). Human adipose-derived stromal/stem cells demonstrate short-lived persistence after implantation in both an immunocompetent and an immunocompromised murine model. Stem Cell Res. Ther..

[bib2] Angelotti M.L., Ronconi E., Ballerini L., Peired A., Mazzinghi B., Sagrinati C., Parente E., Gacci M., Carini M., Rotondi M. (2012). Characterization of renal progenitors committed toward tubular lineage and their regenerative potential in renal tubular injury. Stem Cells.

[bib3] Asderakis A., Dyer P., Augustine T., Worthington J., Campbell B., Johnson R.W. (2001). Effect of cold ischemic time and HLA matching in kidneys coming from “young” and “old” donors: do not leave for tomorrow what you can do tonight. Transplantation.

[bib4] Bates M.L., Fulmer B.R., Farrell E.T., Drezdon A., Pegelow D.F., Conhaim R.L., Eldridge M.W. (2012). Hypoxia recruits intrapulmonary arteriovenous pathways in intact rats but not isolated rat lungs. J. Appl. Physiol..

[bib5] Bentzon J.F., Stenderup K., Hansen F., Schroder H., Abdallah B., Jensen T., Kassem M. (2005). Tissue distribution and engraftment of human mesenchymal stem cells immortalized by human telomerase reverse transcriptase gene. Biochem. Biophys. Res. Commun..

[bib6] Bruno S., Grange C., Collino F., Deregibus M.C., Cantaluppi V., Biancone L., Tetta C., Camussi G. (2012). Microvesicles derived from mesenchymal stem cells enhance survival in a lethal model of acute kidney injury. PloS One.

[bib7] Bruno S., Grange C., Deregibus M.C., Calogero R.A., Saviozzi S., Collino F., Morando L., Falda M., Bussolati B., Tetta C. (2009). Mesenchymal stem cell-derived microvesicles protect against acute tubular injury. J. Am. Soc. Nephrol..

[bib8] Bussolati B., Bruno S., Grange C., Buttiglieri S., Deregibus M.C., Cantino D., Camussi G. (2005). Isolation of renal progenitor cells from adult human kidney. Am. J. Pathol..

[bib9] Bussolati B., Camussi G. (2015). Therapeutic use of human renal progenitor cells for kidney regeneration. Nat. Rev. Nephrol..

[bib100] Buehler P.W., D’Agnillo F., Schaer D.J. (2010). Hemoglobin-based oxygen carriers: from mechanisms of toxicity and clearance to rational drug design. Trends Mol. Med..

[bib10] De Paul M.A., Palmer M., Lang B.T., Cutrone R., Tran A.P., Madalena K.M., Bogaerts A., Hamilton J.A., Deans R.J., Mays R.W., Busch S.A., Silver J. (2015). Intravenous multipotent adult progenitor cell treatment decreases inflammation leading to functional recovery following spinal cord injury. Sci. Rep..

[bib11] Deliolanis N.C., Ale A., Morscher S., Burton N.C., Schaefer K., Radrich K., Razansky D., Ntziachristos V. (2014). Deep-tissue reporter-gene imaging with fluorescence and optoacoustic tomography: a performance overview. Mol. Imaging Biol..

[bib12] Donizetti-Oliveira C., Semedo P., Burgos-Silva M., Cenedeze M.A., Malheiros D.M.A.C., Reis M.A., Pacheco-Silva A., Câmara N.O.S. (2012). Adipose tissue-derived stem cell treatment prevents renal disease progression. Cell Transplant..

[bib13] Durand E., Chaumet-Riffaud P., Grenier N. (2011). Functional Renal Imaging: New Trends in Radiology and Nuclear Medicine, (Seminars in Nuclear Medicine).

[bib14] Ebrahimi B., Textor S.C., Lerman L.O. (2013). Renal relevant radiology: renal functional magnetic resonance imaging. Clin. J. Am. Soc. Nephrol..

[bib15] Egger C., Cannet C., Gérard C., Debon C., Stohler N., Dunbar A., Tigani B., Li J., Beckmann N. (2015). Adriamycin-induced nephropathy in rats: functional and cellular effects characterized by MRI. J. Magn. Reson. Imaging.

[bib16] Eisner C., Faulhaber-Walter R., Wang Y., Leelahavanichkul A., Yuen P.S., Mizel D., Star R.A., Briggs J.P., Levine M., Schnermann J. (2010). Major contribution of tubular secretion to creatinine clearance in mice. Kidney Int..

[bib101] Ermolayev V., Dean-Ben X.L., Mandal S., Ntziachristos V., Razansky D. (2016). Simultaneous visualization of tumour oxygenation, neovascularization and contrast agent perfusion by real-time three-dimensional optoacoustic tomography. Eur. Radiol..

[bib17] Feng G., Zhang J., Li Y., Nie Y., Zhu D., Wang R., Liu J., Gao J., Liu N., He N. (2016). IGF-1C domain–modified hydrogel enhances cell therapy for AKI. J. Am. Soc. Nephrol..

[bib18] Feng Z., Ting J., Alfonso Z., Strem B.M., Fraser J.K., Rutenberg J., Kuo H.-C., Pinkernell K. (2010). Fresh and cryopreserved, uncultured adipose tissue-derived stem and regenerative cells ameliorate ischemia-reperfusion-induced acute kidney injury. Nephrol. Dial. Transplant..

[bib19] Fischer K., Meral F.C., Zhang Y., Vangel M.G., Jolesz F.A., Ichimura T., Bonventre J.V. (2016). High-resolution renal perfusion mapping using contrast-enhanced ultrasonography in ischemia-reperfusion injury monitors changes in renal microperfusion. Kidney Int..

[bib20] Fischer U.M., Harting M.T., Jimenez F., Monzon-Posadas W.O., Xue H., Savitz S.I., Laine G.A., Cox C.S. (2009). Pulmonary passage is a major obstacle for intravenous stem cell delivery: the pulmonary first-pass effect. Stem Cells Dev..

[bib21] Fuente Mora C., Ranghini E., Bruno S., Bussolati B., Camussi G., Wilm B., Edgar D., Kenny S., Murray, P E. (2012). Differentiation of podocyte and proximal tubule-like cells from a mouse kidney-derived stem cell line. Stem Cells Dev..

[bib22] Geng Y., Zhang L., Fu B., Zhang J., Hong Q., Hu J., Li D., Luo C., Cui S., Zhu F. (2014). Mesenchymal stem cells ameliorate rhabdomyolysis-induced acute kidney injury via the activation of M2 macrophages. Stem Cell Res. Ther..

[bib23] Gibney R.N., Sever M.S., Vanholder R.C. (2014). Disaster nephrology: crush injury and beyond. Kidney Int..

[bib24] Gooch A., Westenfelder C. (2016). Modified hydrogels to enhance cellular therapy for AKI: a translational challenge. J. Am. Soc. Nephrol..

[bib25] Grange C., Moggio A., Tapparo M., Porta S., Camussi G., Bussolati B. (2014). Protective effect and localization by optical imaging of human renal CD133+ progenitor cells in an acute kidney injury model. Physiol. Rep..

[bib26] Grange C., Tapparo M., Bruno S., Chatterjee D., Quesenberry P.J., Tetta C., Camussi G. (2014). Biodistribution of mesenchymal stem cell-derived extracellular vesicles in a model of acute kidney injury monitored by optical imaging. Int. J. Mol. Med..

[bib27] Harari-Steinberg O., Metsuyanim S., Omer D., Gnatek Y., Gershon R., Pri-Chen S., Ozdemir D.D., Lerenthal Y., Noiman T., Ben-Hur H. (2013). Identification of human nephron progenitors capable of generation of kidney structures and functional repair of chronic renal disease. EMBO Mol. Med..

[bib28] Herrler T., Wang H., Tischer A., Bartenstein P., Jauch K.-W., Guba M., Diemling M., Nimmon C., Hacker M. (2012). 99mTc-MAG3 scintigraphy for the longitudinal follow-up of kidney function in a mouse model of renal ischemia-reperfusion injury. EJNMMI Res..

[bib29] Heslop J.A., Hammond T.G., Santeramo I., Piella A.T., Hopp I., Zhou J., Baty R., Graziano E.I., Marco B.P., Caron A. (2015). Concise review: workshop review: understanding and assessing the risks of stem cell-based therapies. Stem Cells Transl. Med..

[bib30] Imberti B., Tomasoni S., Ciampi O., Pezzotta A., Derosas M., Xinaris C., Rizzo P., Papadimou E., Novelli R., Benigni A. (2015). Renal progenitors derived from human iPSCs engraft and restore function in a mouse model of acute kidney injury. Sci. Rep..

[bib31] Inai H., Kawai K., Ikeda A., Ando S., Kimura T., Oikawa T., Onozawa M., Miyazaki J., Uchida K., Nishiyama H. (2013). Risk factors for chronic kidney disease after chemotherapy for testicular cancer. Int. J. Urol..

[bib32] James M.L., Gambhir S.S. (2012). A molecular imaging primer: modalities, imaging agents, and applications. Physiol. Rev..

[bib33] Jathoul A.P., Laufer J., Ogunlade O., Treeby B., Cox B., Zhang E., Johnson P., Pizzey A.R., Philip B., Marafioti T. (2015). Deep in vivo photoacoustic imaging of mammalian tissues using a tyrosinase-based genetic reporter. Nat. Photonics.

[bib34] Jerome S.N., Akimitsu T., Korthuis R.J. (1994). Leukocyte adhesion, edema, and development of postischemic capillary no-reflow. Am. J. Physiol.-Heart Circ. Physiol..

[bib36] Kean T.J., Lin P., Caplan A.I., Dennis J.E. (2013). MSCs: delivery routes and engraftment, cell-targeting strategies, and immune modulation. Stem Cells Int..

[bib37] Kunter U., Rong S., Boor P., Eitner F., Müller-Newen G., Djuric Z., van Roeyen C.R., Konieczny A., Ostendorf T., Villa L. (2007). Mesenchymal stem cells prevent progressive experimental renal failure but maldifferentiate into glomerular adipocytes. J. Am. Soc. Nephrol..

[bib38] Lai C.P., Mardini O., Ericsson M., Prabhakar S., Maguire C.A., Chen J.W., Tannous B.A., Breakefield X.O. (2014). Dynamic biodistribution of extracellular vesicles in vivo using a multimodal imaging reporter. ACS Nano.

[bib39] Lassailly F., Griessinger E., Bonnet D. (2010). “Microenvironmental contaminations” induced by fluorescent lipophilic dyes used for noninvasive in vitro and in vivo cell tracking. Blood.

[bib40] Li W., Jiang H., Feng J.-M. (2012). Isogenic mesenchymal stem cells transplantation improves a rat model of chronic aristolochic acid nephropathy via upregulation of hepatic growth factor and downregulation of transforming growth factor β1. Mol. Cell. Biochem..

[bib41] Lovering A.T., Elliott J.E., Beasley K.M., Laurie S.S. (2010). Pulmonary pathways and mechanisms regulating transpulmonary shunting into the general circulation: an update. Injury.

[bib42] Lu Y., Darne C.D., Tan I.-C., Wu G., Wilganowski N., Robinson H., Azhdarinia A., Zhu B., Rasmussen J.C., Sevick-Muraca E.M. (2013). In vivo imaging of orthotopic prostate cancer with far-red gene reporter fluorescence tomography and in vivo and ex vivo validation. J. Biomed. Opt..

[bib43] Mahoney M., Sorace A., Warram J., Samuel S., Hoyt K. (2014). Volumetric contrast-enhanced ultrasound imaging of renal perfusion. J. Ultrasound Med..

[bib44] Maldiney T., Bessière A., Seguin J., Teston E., Sharma S.K., Viana B., Bos A.J., Dorenbos P., Bessodes M., Gourier D. (2014). The in vivo activation of persistent nanophosphors for optical imaging of vascularization, tumours and grafted cells. Nat. Mater..

[bib45] Malliaras K., Marban E. (2011). Cardiac cell therapy: where we’ve been, where we are, and where we should be headed. Br. Med. Bull..

[bib46] Mandal S., Dean-Ben X.L., Burton N.C., Razansky D. (2015). Extending biological imaging to the fifth dimension: evolution of volumetric small animal multispectral optoacoustic tomography. IEEE Pulse.

[bib47] Mao S., Xu H., Zou L., Xu G., Wu Z., Ding Q., Jiang H. (2014). Estrogen preserves split renal function in a chronic complete unilateral ureteral obstruction animal model. Exp. Ther. Med..

[bib48] Meleshina A., Cherkasova E., Shirmanova M., Khrapichev A., Dudenkova V., Zagaynova E. (2015). Modern techniques for stem cells in vivo imaging (Review). Med. Technol. Med./Sovrem. Tehnol. Med..

[bib49] Morigi M., Rota C., Montemurro T., Montelatici E., Lo Cicero V., Imberti B., Abbate M., Zoja C., Cassis P., Longaretti L. (2010). Life-sparing effect of human cord blood-mesenchymal stem cells in experimental acute kidney injury. Stem Cells.

[bib50] Moroz M.A., Serganova I., Zanzonico P., Ageyeva L., Beresten T., Dyomina E., Burnazi E., Finn R.D., Doubrovin M., Blasberg R.G. (2007). Imaging hNET reporter gene expression with 124I-MIBG. J. Nucl. Med..

[bib51] Murray P.A., Woolf A.S. (2014). Using stem and progenitor cells to recapitulate kidney development and restore renal function. Curr. Opin. Organ Transplant..

[bib53] Okusa M.D., Chertow G.M., Portilla D., Nephrologyo.t.A.S.o A.K.I.A.G. (2009). The nexus of acute kidney injury, chronic kidney disease, and World Kidney Day 2009. Clin. J. Am. Soc. Nephrol..

[bib54] Papazova D.A., Oosterhuis N.R., Gremmels H., van Koppen A., Joles J.A., Verhaar M.C. (2015). Cell-based therapies for experimental chronic kidney disease: a systematic review and meta-analysis. Dis. Models Mech..

[bib55] Pereira S.M., Herrmann A., Moss D., Poptani H., Williams S.R., Murray P., Taylor A. (2016). Evaluating the effectiveness of transferrin receptor-1 (TfR1) as a magnetic resonance reporter gene. Contrast Media Mol. Imaging.

[bib56] Pereira S.M., Moss D., Williams S.R., Murray P., Taylor A. (2015). Overexpression of the MRI reporter genes ferritin and transferrin receptor affect iron homeostasis and produce limited contrast in mesenchymal stem cells. Int. J. Mol. Sci..

[bib57] Pereira S.M., Williams S.R., Murray P., Taylor A. (2016). MS-1 magA revisiting its efficacy as a reporter gene for MRI. Mol. Imaging.

[bib58] Progatzky F., Dallman M.J., Celso C.L. (2013). From seeing to believing: labelling strategies for in vivo cell-tracking experiments. Interface Focus.

[bib59] Qi S., Wu D. (2013). Bone marrow-derived mesenchymal stem cells protect against cisplatin-induced acute kidney injury in rats by inhibiting cell apoptosis. Int. J. Mol. Med..

[bib60] Rodrigo R., Trujillo S., Bosco C. (2006). Biochemical and ultrastructural lung damage induced by rhabdomyolysis in the rat. Exp. Biol. Med..

[bib61] Ronconi E., Sagrinati C., Angelotti M.L., Lazzeri E., Mazzinghi B., Ballerini L., Parente E., Becherucci F., Gacci M., Carini M. (2009). Regeneration of glomerular podocytes by human renal progenitors. J. Am. Soc. Nephrol..

[bib62] Rosado-de-Castro P.H., Pimentel-Coelho P.M., Gutfilen B., Lopes de Souza S.A., De Freitas G.R., Mendez-Otero R., Barbosa da Fonseca L.M. (2014). Radiopharmaceutical stem cell tracking for neurological diseases. BioMed. Res. Int..

[bib63] Scarfe L., Rak-Raszewska A., Geraci S., Darssan D., Sharkey J., Huang J., Burton N.C., Mason D., Ranjzad P., Kenny S. (2015). Measures of kidney function by minimally invasive techniques correlate with histological glomerular damage in SCID mice with adriamycin-induced nephropathy. Sci. Rep..

[bib64] Schock-Kusch D., Xie Q., Shulhevich Y., Hesser J., Stsepankou D., Sadick M., Koenig S., Hoecklin F., Pill J., Gretz N. (2011). Transcutaneous assessment of renal function in conscious rats with a device for measuring FITC-sinistrin disappearance curves. Kidney Int..

[bib65] Semedo P., Donizetti-Oliveira C., Burgos-Silva M., Cenedeze M.A., Malheiros D.M.A.C., Pacheco-Silva A., Câmara N.O.S. (2010). Bone marrow mononuclear cells attenuate fibrosis development after severe acute kidney injury. Lab. Invest..

[bib66] Sullivan J.C., Wang B., Boesen E.I., D’Angelo G., Pollock J.S., Pollock D.M. (2009). Novel use of ultrasound to examine regional blood flow in the mouse kidney. Am. J. Physiol.-Ren. Physiol..

[bib67] Sun Y., Yu H., Zheng D., Cao Q., Wang Y., Harris D., Wang Y. (2011). Sudan black B reduces autofluorescence in murine renal tissue. Arch. Pathol. Lab. Med..

[bib68] Taruttis A., Morscher S., Burton N.C., Razansky D., Ntziachristos V. (2012). Fast multispectral optoacoustic tomography (MSOT) for dynamic imaging of pharmacokinetics and biodistribution in multiple organs. PLoS One.

[bib69] Taruttis A., Ntziachristos V. (2015). Advances in real-time multispectral optoacoustic imaging and its applications. Nat. Photonics.

[bib70] Taylor A., Herrmann A., Moss D., Sée V., Davies K., Williams S.R., Murray P. (2014). Assessing the efficacy of nano-and micro-sized magnetic particles as contrast agents for MRI cell tracking. PloS One.

[bib71] Taylor A., Wilson K.M., Murray P., Fernig D.G., Lévy R. (2012). Long-term tracking of cells using inorganic nanoparticles as contrast agents: are we there yet?. Chem. Soc. Rev..

[bib72] Tirotta I., Dichiarante V., Pigliacelli C., Cavallo G., Terraneo G., Bombelli F.B., Metrangolo P., Resnati G. (2014). 19F Magnetic Resonance Imaging (MRI): from design of materials to clinical applications. Chem. Rev..

[bib73] To H., Ohdo S., Shin M., Uchimaru H., Yukawa E., Higuchi S., Fujimura A., Kobayashi E. (2003). Dosing time dependency of doxorubicin-induced cardiotoxicity and bone marrow toxicity in rats. J. Pharm. Pharmacol..

[bib74] Tögel F., Yang Y., Zhang P., Hu Z., Westenfelder C. (2008). Bioluminescence imaging to monitor the in vivo distribution of administered mesenchymal stem cells in acute kidney injury. Am. J. Physiol.-Ren. Physiol..

[bib75] Toyohara T., Mae S.-I., Sueta S.-I., Inoue T., Yamagishi Y., Kawamoto T., Kasahara T., Hoshina A., Toyoda T., Tanaka H. (2015). Cell therapy using human induced pluripotent stem cell-derived renal progenitors ameliorates acute kidney injury in mice. Stem Cells Transl. Med..

[bib76] Velde G.V., Himmelreich U., Neeman M. (2013). Reporter gene approaches for mapping cell fate decisions by MRI: promises and pitfalls. Contrast Media Mol. Imaging.

[bib77] Wakabayashi H., Werner R.A., Hayakawa N., Javadi M.S., Chen X., Herrman K., Rowe S.P., Lapa C., Higuchi T. (2016). Initial preclinical evaluation of 18F-fluorodeoxysorbitol PET as a novel functional renal imaging agent. J. Nucl. Med..

[bib78] Wang H.-J., Varner A., AbouShwareb T., Atala A., Yoo J.J. (2012). Ischemia/reperfusion-induced renal failure in rats as a model for evaluating cell therapies. Ren. Fail..

[bib79] Wang Y.-X.J., Yan S.-X. (2008). Biomedical imaging in the safety evaluation of new drugs. Lab. Anim..

[bib102] Wang L.V., Hu S. (2012). Photoacoustic tomography: in vivo imaging from organelles to organs. Science.

[bib80] Winter P.M. (2014). Perfluorocarbon nanoparticles: evolution of a multimodality and multifunctional imaging agent. Scientifica.

[bib81] Yang H.-Y., Chen P.-C., Wang J.-D. (2014). Chinese herbs containing aristolochic acid associated with renal failure and urothelial carcinoma: a review from epidemiologic observations to causal inference. BioMed. Res. Int..

[bib82] Zhuo, W., Liao, L., Fu, Y., Xu, T., Wu, W., Yang, S., Tan, J., 2013. Efficiency of endovenous versus arterial administration of mesenchymal stem cells for ischemia-reperfusion–induced renal dysfunction in rats. In: Proceedings of the Transplantation, Elsevier. pp. 503–510 10.1016/j.transproceed.2012.07.162 23498785

[bib83] Zöllner F.G., Schock-Kusch D., Bäcker S., Neudecker S., Gretz N., Schad L.R. (2013). Simultaneous measurement of kidney function by dynamic contrast enhanced MRI and FITC-sinistrin clearance in rats at 3 T: initial results. PloS One.

